# Evaluation of calcium and folic acid supplementation in prenatal care in São Paulo

**DOI:** 10.1590/S1516-31802010000600003

**Published:** 2010-12-02

**Authors:** Camila Atallah Pontes da Silva, Carolina Atallah Pontes da Silva, Álvaro Nagib Atallah, Nelson Sass, Eliane Terezinha Rocha Mendes, Sérgio Peixoto

**Affiliations:** I MD. Otorhinolaryngologist, Faculdade de Medicina da Fundação ABC (FMABC), Santo André, São Paulo, Brazil.; II MD. Dermatologist, Faculdade de Medicina de São José do Rio Preto (Famerp), São José do Rio Preto, São Paulo, Brazil.; III MD, PhD. Titular professor of the Discipline of Emergency and Evidence-Based Medicine, Universidade Federal de São Paulo — Escola Paulista de Medicina (Unifesp-EPM), São Paulo, São Paulo, Brazil.; IV MD, PhD. Adjunct professor in the Discipline of Pathological and Surgical Obstetrics, Universidade Federal de São Paulo — Escola Paulista de Medicina (Unifesp-EPM), São Paulo, São Paulo, Brazil.; V MD. Attending physician in the Discipline of Gynecology and Obstetrics, Faculdade de Medicina da Fundação ABC (FMABC), Santo André, São Paulo, Brazil.; VI MD PhD. Titular professor of the Discipline of Gynecology and Obstetrics, Faculdade de Medicina da Fundação ABC (FMABC), Santo André, São Paulo, Brazil.

**Keywords:** Calcium, Folic acid, Neural tube defects, Pre-eclampsia, Dietary supplements, Cálcio, Ácido fólico, Defeitos do tubo neural, Pré-eclâmpsia, Suplementos dietéticos

## Abstract

**CONTEXT AND OBJECTIVE:**

Preeclampsia and neural tube defects can be prevented during pregnancy. Today, there is level I evidence showing that calcium supplementation during pregnancy may prevent preeclampsia and that use of folic acid may prevent neural tube defects. The aim here was to evaluate the proportion of patients undergoing prenatal follow-up who had received a prescription for calcium and/or folic acid supplementation, and their adherence to the use of these two substances.

**DESIGN AND SETTING:**

Cross-sectional study at two hospitals in the Greater São Paulo region, Brazil (Faculdade de Medicina da Fundação ABC, Santo André, and “Dr. Mário de Moraes Altenfelder Silva” Municipal Teaching and Maternity Hospital, Vila Nova Cachoeirinha).

**METHODS:**

Early primigravidae, late primigravidae and pregnant women with chronic hypertension, diabetes mellitus or kidney disease who had already had their first prenatal consultation were included.

**RESULTS:**

Out of 250 pregnant women interviewed, 10.40% had received a prescription for calcium supplementation and 80.76% of them reported taking it in tablet form. Regarding folic acid, 48% of the women said that they had received a prescription for this and 64.16% reported that they had started to use it during the periconceptional period.

**CONCLUSIONS:**

Calcium supplementation and periconceptional use of folic acid seem not to be prescribed routinely by physicians. This should motivate the implementation of educational programs for obstetricians on the use of interventions based on the best available evidence.

## RESUMO

**CONTEXTO E OBJETIVO:**

Pré-eclâmpsia e defeitos no tubo neural podem ser prevenidas na gravidez. Atualmente há evidência de nível I de que a suplementação de cálcio durante a gestação pode prevenir pré-eclâmpsia, e que o uso de ácido fólico pode prevenir defeitos no tubo neural. O objetivo foi avaliar a proporção de pacientes em acompanhamento de pré-natal que recebem prescrição de suplementação de cálcio e/ou ácido fólico, bem como a proporção das pacientes prescritas que aderem ao uso das duas substâncias.

**TIPO DE ESTUDO E LOCAL:**

Estudo transversal em dois hospitais da grande São Paulo (Faculdade de Medicina da Fundação ABC, Santo André, e Hospital Municipal Maternidade Escola “Dr. Mário de Moraes Altenfelder Silva”, Vila Nova Cachoeirinha).

**MÉTODOS:**

Foram incluídas primigestas precoces e tardias e gestantes hipertensas crônicas, e/ou com diabetes mellitus e nefropatas com a primeira consulta de pré-natal já realizada.

**RESULTADOS:**

Das 250 gestantes entrevistadas, 10,4% receberam prescrição de suplementação de cálcio, sendo que 80,76% responderam fazer uso de cálcio na forma de comprimidos. Em relação ao ácido fólico, 48% responderam ter recebido prescrição de suplementos contendo a substância, sendo que 64.16% começaram o uso dentro do período periconcepcional.

**CONCLUSÕES:**

A suplementação de cálcio e o uso periconcepcional de ácido fólico parecem não ser prescritos de forma rotineira pelos médicos, o que deve motivar a adoção de programas educacionais para obstetras sobre a utilização de intervenções baseadas na melhor evidência disponível.

## INTRODUCTION

Pregnancy is a period of many maternal transformations, including intense functional and metabolic modifications. Adequate care for pregnant women is of prime importance, starting at the beginning of pregnancy, when the implementation of specific interventions can effectively reduce the risk of disorders such as preeclampsia and neural tube defects.

Preeclampsia is characterized by arterial hypertension and significant proteinuria, with onset generally after the 20^th^ week of pregnancy.^1^ It is still an important cause of maternal mortality, especially in developing countries,^2^ and it affects 6 to 10% of all pregnant women, with higher incidence among adolescents and older nulliparae.^1^ It may rapidly progress to a severe form that causes damage to the central nervous system, eyes, kidneys and heart and may also jeopardize the fetus. When it is accompanied by convulsions, it is called eclampsia.^1^

Calcium supplementation for pregnant women, as an intervention to reduce the risk of developing preeclampsia, has been investigated over the course of the years. Clinical and epidemiological studies^3-7^ have confirmed that there is an inverse relationship between calcium intake and hypertensive disorders of pregnancy. This has resulted in the hypothesis that increased calcium intake during pregnancy would reduce the incidence of hypertension and preeclampsia, especially among women with low calcium intake. This hypothesis has been tested in several randomized studies since the end of the 1980s. A systematic review showed that calcium supplementation among high-risk women had beneficial effects, with reductions in the incidence of preeclampsia and eclampsia.^8^

Neural tube defects, which include anencephaly, spina bifida and encephalocele, occur approximately one month after fertilization.^9^ During pregnancy, there is greater requirement for folic acid, since the growth of the fetus causes an increase in the number of cells, which are dividing rapidly.^10^

With one exception,^11^ all studies carried out since 1980 have reported that the risk of neural tube defects was lower among women with increased folic acid intake and those who received multivitamin or folic acid supplementation during the periconceptional period.^12-19^ The overall recurrence rate of neural tube defects in subsequent pregnancies is estimated to be 4 to 5%.^20^ It must also be emphasized that, in the absence of folate supplementation during pregnancy, more than one third of women develop below-normal postpartum serum folate levels, and 3.4% present megaloblastic anemia.^21^

Thus, it becomes essential to advise women to use calcium and folic acid supplementation during pregnancy and the periconceptional period, respectively, as a means of reducing the risks of preeclampsia and neural tube defects. This is especially important for primigravidae and women with a history of neural tube defect in previous pregnancies.

Today, there is level IA evidence (from systematic reviews with meta-analysis)^7^ that the use of calcium during pregnancy reduces the risk of preeclampsia, and that folic acid supplementation reduces the risk of neural tube defects.^9,10^ These practices are recommended in the clinical guidelines of the World Health Organization (WHO).^22^

Therefore, taking into account the levels of evidence that support the systematic use of these recommendations, we decided to assess to what extent these practices were being implemented in routine prenatal clinical care, among a population of pregnant women in the Greater São Paulo region.

## OBJECTIVE

To evaluate 1) the proportions of patients undergoing prenatal follow-up who received a prescription for supplementation of calcium or folic acid, and 2) the proportion of patients receiving such prescriptions who adhered to the use of these two substances.

## MATERIAL AND METHODS

### Type of study

A cross-sectional study was conducted to quantitatively and qualitatively assess the prescription of calcium and folic acid to pregnant women.

### Location of the study

The study was carried out in two teaching hospitals within the Brazilian National Health System (Sistema Único de Saúde, SUS) in the Greater São Paulo region between May 2004 and May 2005. These institutions were Faculdade de Medicina da Fundação ABC (FMABC) and “Dr. Mário de Moraes Altenfelder Silva” Municipal Teaching and Maternity Hospital, Vila Nova Cachoeirinha. The women involved in this study were of low socioeconomic and educational level. The project was approved by the Research Ethics Committees of both institutions.

### Inclusion criteria

The study included early primigravidae (under 16 years old), late primigravidae (over 35 years old) and pregnant women with chronic hypertension (CH), diabetes mellitus (DM) or kidney disease, who had already had been to at least one prenatal care consultation.

### Exclusion criteria

Patients with cognitive deficit who might have compromised the data collection were excluded.

### Considerations regarding adequate calcium supplementation

Current evidence^8^ indicates that 500 to 2000 mg of calcium per day is sufficient during pregnancy, to reduce the risk of preeclampsia. The upper limit of this range (2000 mg) corresponds to a daily intake of approximately eight 200-ml glasses of milk (skimmed or whole milk), or 200 grams of cheese.

### Considerations regarding adequate folic acid supplementation

The WHO clinical guidelines recommend a daily dose of 0.4 mg of folic acid per day for women of fertile age during the periconceptional period.^22^

### Sample size

Assuming a statistical power of 90%, a proportion of 30% of pregnant women making use of calcium and/or folic acid, and a sample error of 6%, with a 95% confidence interval (CI) (α of 0.05), the predicted sample size was 233 pregnant women.

### Data collection

Women attending the antenatal clinic or in the postpartum ward of the two hospitals were approached by the researchers who explained the study objectives and invited them to participate voluntarily. After the women had signed the informed consent form, they were interviewed orally, using a semi-structured questionnaire (Annex 1). The interviews were individual and lasted an average of 10 minutes each. After completion, the questionnaire was put inside a plain, unmarked envelope, sealed and deposited in a ballot-box for subsequent analysis, without patient identification.

## RESULTS

A total of 250 pregnant women were invited to participate in the study at the two centers and all accepted. Their ages ranged from 13 to 45 years, with a mean of 25.9 years. Sixty-eight of these women (27.20%) were primigravidae. The mean number of pregnancies was 2.8. Among the participants, 19.20% had CH, 6.00% had DM, 3.60% kidney disease, 45.60% had a positive family history of CH, 30.12% had a positive family history of preeclampsia (PE) and 2% had had multiple gestations.

Out of the total number of patients, 26 women (10.40%) received a recommendation to supplement their calcium intake, and 21 of these (80.76%) said that they had taken calcium in the form of tablets. Among the whole study sample, 108 women (43.20%) said that they had been advised by their physician to consume a greater quantity of calcium-rich foods, and 75% of them said that they had followed or were following the recommended diet. Among those who had followed or were following the diet, 57 women (70.37%) stated that they were consuming at least two glasses of milk per day, which corresponded to approximately 500 mg of calcium, i.e. the minimum recommended dose for adequate calcium supplementation.^7^

Among the primigravidae, six women (8.82%) received a calcium prescription. Also among the primigravidae, nine women (13.24%) presented CH, three women (4.41%) presented DM and 13 women (19.12%) had a positive family history of CH.

Among the 182 multiparae, 20 women (11.00%) received calcium prescription and, of these, five women presented a positive family history of CH, two had personal histories of preeclampsia, three women had CH. Among the multiparae, 120 women (65.93%) had received prescriptions for vitamins. Among the patients who had received prescriptions, 21 women (80.77%) said that they had used calcium and 88 women (73.33%) said that they had used folic acid.

Regarding the prevention of neural tube defects, 120 women (48%) said that they had received prescriptions for vitamins: 32 (26.70%) multivitamins and 88 (73.30%), exclusively folic acid. The average number of daily tablets was 1.3, while the exact folic acid dose could not be determined, since almost none of the patients knew this information. Most of the women (77), said that they had started taking the vitamins during the periconceptional period, while 43 women started at two months of gestation, which is the limit for obtaining the protective effect from the substance, according to a systematic review.^9^

When asked about the importance of folic acid supplementation at the start of pregnancy, 80 women (66.76%) said that they had been informed about this by their physician during prenatal visits.

## DISCUSSION

Calcium supplementation during pregnancy has been shown to be effective in reducing the incidence and severity of gestational hypertensive disease among high-risk women and among populations with low calcium intake.^23^ This intervention is relatively inexpensive and is easily available, since it can be achieved in the form of generic tablets or through increased intake of calcium-rich foods.

The results from our study indicate that only a small percentage (10.40%) of the patients interviewed, mostly women at high risk of preeclampsia and undergoing prenatal care, actually received a prescription for calcium supplementation. Likewise, less than half (43.20%) were counseled to include calcium-rich foods in their diets.

Periconceptional folic acid supplementation, i.e. before the pregnancy and during the first two months of gestation, has a protective effect against neural tube defects.^9^ In this study, less than half of the pregnant women interviewed reported having received a prescription for this vitamin. Nevertheless, among those who did receive such a prescription, most took the supplement within the periconceptional period. Out of the total number of patients studied, more than half of them reported receiving medical advice regarding the importance of using folic acid at the start of the pregnancy.

The present study was conducted on a significant number of patients, thereby enabling assessment of daily clinical practice regarding the use of medical evidence that is very widely disseminated. It must, however, be borne in mind that this study was based on a questionnaire that has not undergone validation, which may have caused systematic error. Nonetheless, because the sample was obtained from different hospitals, it is reasonable to infer that this scenario reflects the reality of the population of interest for the present study. Moreover, it is important to consider that this study involved women of low educational level, which may have contributed towards their difficulty in understanding some questions, especially regarding the names and dosages of the medicines that they used. There are no similar questionnaires in the literature, which impedes comparisons with other publications. This implies that validation is needed in order to make future comparisons.

There are difficulties in achieving correct and effective supplementation of both calcium and folic acid. Consequently, many opportunities for preventing preeclampsia and the associated maternal-fetal morbidity and mortality, as well as severe fetal malformations, are lost. One way to help solve this problem would be fortification of certain foods (in addition to wheat flour), with these two substances, which would facilitate access to them for all reproductive-age and pregnant women. However, the effects of increased doses of calcium and folic acid on the remainder of the population are still unknown and would need to be studied. Thus, the best strategy would be to adopt interventions of an educational nature. Creation and dissemination of guidelines on this matter among health professionals and the lay population are vitally important. These are simple and inexpensive ways of improving the health of pregnant women, and they improve the prospects of good health for the present and future generations.

## CONCLUSION

Despite the available level I evidence, neither calcium during pregnancy nor folic acid during the periconcepcional period seem to be routinely prescribed by physicians, according to the report of the women interviewed.

The present study indicates that there is a need for further assessment of the knowledge and daily medical practice of physicians, regarding prescription of calcium and folic acid during pregnancy. Physicians should be informed about the urgent need to seek and use the best evidence, which is frequently presented in the form of guidelines or systematic reviews. Additionally, future studies involving participants of other socioeconomic classes and nationalities could provide a better overall view of this matter.

## Annex 1. Questionnaire, freely translated from Portuguese for this article.

**Table:**

Age (completed years) ________ How many times have you already been pregnant? ____________ Do you have high blood pressure? (Y) (N) Confirmed by: file ( ); prescription ( ); doctor at prenatal consultation ( ) Do you have diabetes mellitus? (Y) (N) Confirmed by: file ( ); prescription ( ); doctor ( ); tested at prenatal consultation ( ) Do you have kidney disease? (Y) (N) Confirmed by: file ( ); doctor ( ); urine I ( ); creatinine ( ) Family antecedents of high blood pressure? (Y) (N) Family antecedents of preeclampsia? (Y) (N) Family antecedents of eclampsia? (Y) (N) Multiple gestation? (Y) (N) Has your doctor prescribed the use of oral calcium supplementation? (Y) (N) Are you taking oral calcium? (Y) (N) Has your doctor prescribed increased consumption of food containing calcium? (Y) (N) What? ______________ Are you following the recommended diet? (Y) (N) How many glasses of milk do you drink per day? ___________ How many grams of cheese do you eat per week? ________ Are you taking vitamins on a medical prescription? (Y) (N) What type? – multivitamins ( ) – folic acid ( ) – vitamin B12 ( ) – B complex ( ) What brand? _________________ How many tablets per day? ______________ What is the prescribed dose of folic acid per day? ___________ When did you start to take multivitamin supplementation? (length of gestation) _____________ Have you been informed about the importance of folic acid supplementation during the periconceptional period? (i.e. before pregnancy and during the first two months of gestation) (Y) (N)
